# Chemical Basis of the Fungicidal Activity of Tobacco Extracts against *Valsa mali*

**DOI:** 10.3390/molecules21121743

**Published:** 2016-12-18

**Authors:** Suzhen Duan, Yongmei Du, Xiaodong Hou, Ning Yan, Weijie Dong, Xinxin Mao, Zhongfeng Zhang

**Affiliations:** 1 Tobacco Research Institute of Chinese Academy of Agricultural Sciences, Qingdao 266101, China; duansuzhen2008@126.com (S.D.); duyongmei@caas.cn (Y.D.); houxiaodong@caas.cn (X.H.); yanning@caas.cn (N.Y.); hidwjnhm@163.com (W.D.); mxxcaas@163.com (X.M.); 2 Graduate School of Chinese Academy of Agricultural Sciences, Beijing 100081, China

**Keywords:** tobacco extracts, *Valsa mali*, fungicide activity, chemical components

## Abstract

Under pressure from social criticism and an unclear future, tobacco researchers have begun to seek alternative uses for the product. Here, we present our study on isolating tobacco compounds with fungicidal activity, which could be used as plant-derived pesticides. Using *Valsa mali* as the target fungus, agar plate tests were conducted to evaluate the fungicidal activity of various tobacco extracts, including tobacco leaves extracts prepared with different solvents, extracts of different tobacco cultivars, and samples from different tobacco organs. Fungal growth morphology was used as the criterion to evaluate the fungicidal activity of tobacco extracts. Correlation analyses between the fungicidal activities and the chemical components of tobacco extracts indicated the major chemical constituents with fungicidal activity. Then, the active compounds were isolated and their effects on the ultra-microstructures of *V. mali* was analyzed using scanning- and transmission-electron microscopy. The results suggested that tobacco extracts prepared with solvents of weaker polarity had higher fungicidal activity, and the inhibitory activity of tobacco extracts against *V. mali* was also cultivar dependent. Furthermore, the fungicidal effects of tobacco flower extracts were higher than those of the leaf extracts. Chemical analysis indicated that cembranoids were the main fungicidal substances, which act by destroying the endometrial structure of the fungus. Tobacco cembranoids at 80 μg/mL could completely inhibit the growth of *V.* *mali*, with an EC_50_ value of 13.18 μg/mL. Our study therefore suggests that tobacco leaves and inflorescences are excellent plant resources for the biological control of *V. mali*.

## 1. Introduction

With the increasing strictness of laws banning smoking across the world, the importance of tobacco as a raw cigarette material will be substantially reduced. In order to minimize the economic impact on tobacco farmers, exploration of alternative tobacco usages is of great importance for tobacco agriculture. One such possibility relies on the role of tobacco as a traditional plant-derived pesticide, as nicotine in tobacco is toxic to most herbivore insects. Nicotine pesticides have been regarded as “green pesticides” with high activity and low toxicity [1]. Previously studies have indicated that tobacco contains some useful ingredients such as solanesol and nicotine, which exhibit potent inhibitory activity against *Staphylococcus aureus*, *Bacillus subtilis*, and *Micrococcus lysodeikticus* [2,3]; in addition, tobacco rhizome extract was found to have a high lethal effect on arachnid mites [4]. However, there are limited reports about the inhibitory effects of tobacco-derived compounds against plant pathogenic fungi.

China is the only country that exports over a million tons of apple, with a total output accounting for more than 50% of the world apple production [5]. Apple tree canker (i.e., rot disease of apple) is one type of skin disease caused by *Valsa mali* that can cause serious damage to the orchards in the apple producing areas in China. Recently, this disease has become an important factor restricting China’s apple production and exportation [6]. Synthetic chemical pesticides, such as asomate (tris-*N*-(dimethyldithiocarbamoyl)arsine), were generally used to control apple tree canker. However, these chemical pesticides usually produce residues polluting the environment [6]. Identification of natural pesticides is therefore of great interest. To discover tobacco-derived pesticides for controlling apple tree canker and to find alternative usage of tobacco, this study analyzed the fungicide activity of tobacco extracts, determined the main effective antifungal components, and investigated the possible antifungal mechanism of tobacco extracts.

## 2. Results and Discussion

### 2.1. Fungicidal Activity of Tobacco Leaf Extracts Using Different Solvents and Investigation of Their Main Fungicidal Components

**Figure 1:**
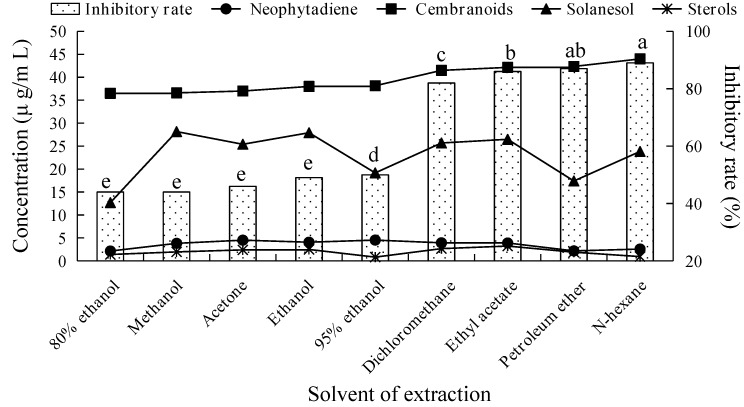
Inhibitory rate against *V. mali* and the concentration of terpenes of tobacco leaf extracts obtained using different solvents. Columns with different letters represent significantly different extraction solvents according to Duncan’s multiple range tests at *p* < 0.05.

We measured the inhibitory rates on *V. mali* growth at 5 mg/mL working concentration of different solvent extracts from the leaves of tobacco cultivar CF87. Figure 1 shows that the inhibitory effects of the various extracts on the growth of *V. mali* differed significantly from each other. The fungicidal activity of the tobacco extract was significantly increased along the decrease of solvent polarity. The inhibitory rates of non-polar solvent (*n*-hexane, petroleum ether) and less-polar solvent (ethyl acetate, dichloromethane) extracts on *V. mali* were above 80%, with notably higher inhibitory activity than that of high polarity solvent extracts (methanol, ethanol, acetone, and their aqueous solutions). According to the different fungicide activities of extracts by various polarity solvent, the main fungicidal components of tobacco leaves were thought to be weakly polar compounds that exhibited higher solubility in less-polar solvents. Therefore, the less-polar solvents (petroleum ether, *n*-hexane, ethyl acetate, and dichloromethane) should be considered the preferred solvents to extract antifungal components from tobacco.

Previous studies showed that plant secondary metabolites terpenes, polyphenols, and flavonoids displayed fungicidal activity [7]. However, polyphenols and flavonoids were not detected in the non-polar and less-polar solvent extracts of CF87 that had strong fungicidal activity in this study. This is probably related to the low solubility of polyphenols and flavonoids in these solvents. Thus, we speculate that polyphenols and flavonoids are not likely to play a major role in the fungicidal activity of tobacco extract.

Figure 1 showed that no obvious correlation was observed between the fungicidal activity and the concentration of solanesol, neophytadiene, and sterols in tobacco extracts obtained by different extraction conditions. In contrast, the fungicidal activity of tobacco extract increased along with the increase of cembranoids concentration. This infers that cembranoids might be the effective antifungal components present in tobacco extracts that display fungicidal activity.

### 2.2. Fungicidal Activity of Tobacco Leaf Extracts from Different Varieties and the Investigation of the Main Antifungal Components

**Figure 2:**
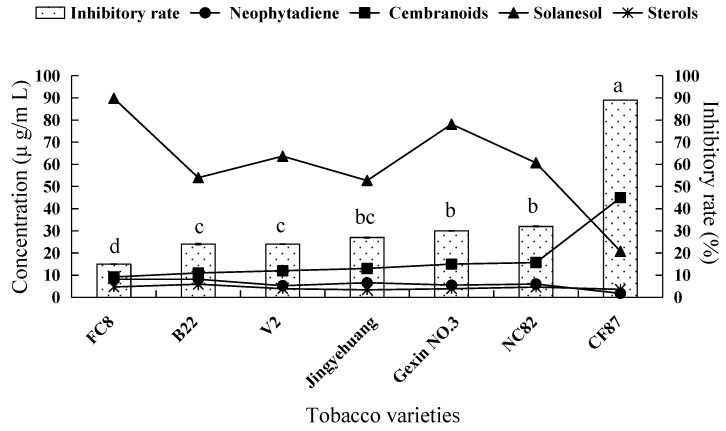
Inhibitory rate against *V. mali* and the concentration of terpenes in different tobacco variety extracts. Columns with different letters represent varieties with significantly different values according to Duncan’s multiple range tests at *p* < 0.05.

The histogram in Figure 2 shows that the inhibitory effects on *V. mali* of *n*-hexane extracts from leaves of different tobacco varieties differed significantly from each other. Among them, the inhibitory activity of extract from tobacco CF87 was the strongest, with an inhibitory rate of 89%. The extracts from tobacco NC82, Gexin NO.3, Jingyehuang, V2, B22, and FC8 showed relatively weaker inhibitory effects on the growth of *V. mali*, with the inhibitory rates of 32%, 30%, 27%, 24%, 24%, and 15%, respectively.

Figure 2 shows the concentration of main antifungal components in 5 mg/mL (working concentration) *n*-hexane extracts of tobacco leaves. We found that the concentration of cembranoids showed a notable increasing trend along the increase of fungicidal activity of different tobacco leaves extracts, and the concentration of neophytadiene and solanesol showed a decreasing trend, whereas the variation trend of sterol concentrations was irregular. From the above analyses, we could infer that neophytadiene, solanesol, and phytosterol did not exert inhibitory effect on the growth of *V. mali*, and that cembranoids might represent the effective antifungal components in tobacco extracts.

### 2.3. Fungicidal Activity and Main Antifungal Components of Tobacco Leaf and Flower Extracts

**Figure 3:**
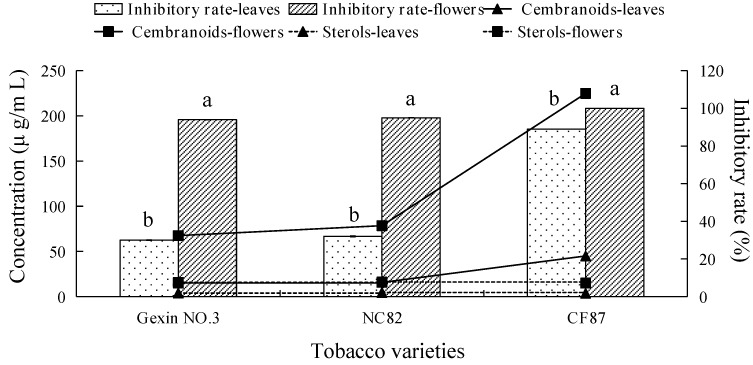
Inhibitory rate against *V. mali* and the concentration of terpenes in tobacco flower and leaf *n*-hexane extracts. Columns with different letters represent organs with significantly different values according to Duncan’s multiple range tests at *p* < 0.05.

The histogram in Figure 3 shows that the inhibitory effects of tobacco flower *n*-hexane extracts were notably higher than those of tobacco leaf extracts from the same tobacco variety.

Chemical composition analyses of the tobacco extracts indicated that polyphenols, flavonoids, neophytadiene, and solanesol were undetable in 5 mg/mL (working concentration) solutions of the different varieties of tobacco flower *n*-hexane extracts. As the fungicidal activity of the tobacco flower extract was significantly higher than that of the tobacco leaves extract, we could infer that these compounds didn’t represent the active chemical ingredients for effective antifungal activity.

Although the concentration of sterols in tobacco flowers extract was significantly higher than that from the leaves, the concentrations of sterols were not correlated with the fungicidal activity of the different flower and leaf extracts (Figure 3). Therefore, we concluded that sterols are not the effective antifungal components in tobacco extracts. On the other hand, the concentration of cembranoids in the flowers extract of different tobacco varieties was 4–8 times higher than that in the leaves extract, and the concentration of cembranoids in the flowers extract of tobacco cultivar CF87, which had the strongest fungicidal activity, was the highest overall, indicating that cembranoids represent the effective antifungal components in tobacco extracts.

Based on the above analyses, cembranoids in tobacco extracts should constitute the major effective antifungal components against *V. mali*. Extensive literature had demonstrated that cembranoids were the main chemical components in tobacco glandular hair exudates [8,9,10]. Because tobacco cembranoids mainly exist at the surface of tobacco leaves and flowers, surface flushing of tobacco leaves or flowers with solvent can be used to obtain high purity cembranoids.

### 2.4. Verification of the Fungicidal Activity of Tobacco Cembranoids Extract on V. mali

**Figure 4:**
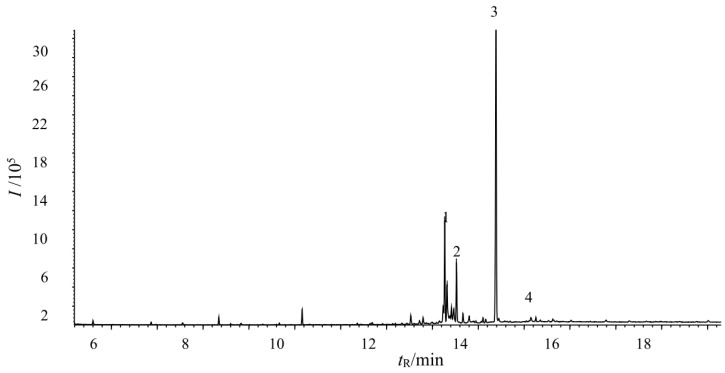
Total ion chromatogram of the tobacco cembranoids extract. Peaks 1, 2, 3, and 4 represent tobacco cembranoids.

Tobacco flowers collected from the flue-cured tobacco variety CF87 were used to extract tobacco cembranoids. Figure 4 showed the total ion chromatograms of cembranoids obtained from tobacco flowers. Peaks 1 and 2 correspond to cembratrienol, peak 3 corresponds to cembratriene diol and peak 4 corresponds to cembratriene triol (Figure 4). By comparison of their mass spectral values with those in the National Institute of Standards and Technology (NIST) database and following quantification by the area normalization method, we calculated that the content of cembranoids in the extract was 92%.

**Figure 5:**
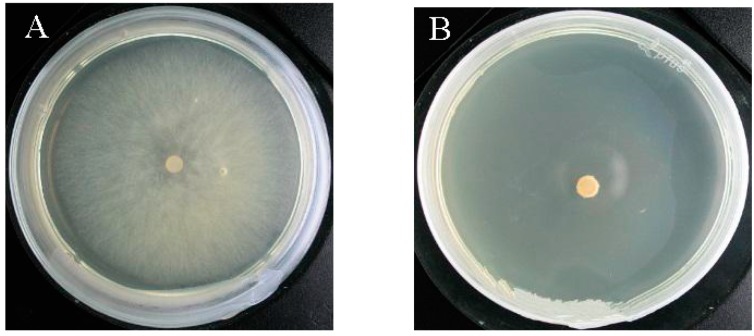
Inhibitory effect of 95% ethanol (**A**) and tobacco cembranoids extract (**B**) on *V. mali*.

Figure 5 shows the antifungal effect of 80 μg/mL (working concentration) tobacco cembranoids extract against *V. mali*. In the control group (Figure 5A), the vegetative hypha grew vigorously; however, in the treatment group (Figure 5B), the growth of vegetative hypha was strongly inhibited with an inhibitory rate of 100%. According to the virulence analyses of tobacco cembranoids extracts against *V.* *mali* at different concentrations (Table 1), the linear regression relationship between the extract concentration and the inhibitory rate was significant, and the regression equation fitted well. The minimal inhibition concentration (MIC) of the tobacco cembranoids extract against *V. mali* was 2.02 μg/mL (working concentration), and the minimal fungicidal concentration (MFC) was 80 μg/mL (working concentration). The EC_50_ of the tobacco cembranoids extract against *V. mali* was 13.18 μg/mL (working concentration). This result further confirmed that the tobacco cembranoids were effective antifungal components.

The above analysis demonstrated that cembranoids were the active antifungal components against *V. mali* in tobacco extracts. Cembranoids belong to the family of 14-membered ring diterpenes and were first isolated from plant oleoresins in tobacco in 1951. Since the 1960s, a series of cembrane-type diterpenoids have been isolated from marine organisms, and over 700 species of cembrane-type diterpenoids have now been isolated from natural sources. These compounds possess novel structures as well as multiple biological activities, including antitumor, antimicrobial, and plant growth regulatory functions. For example, Kennedy et al. [11] studied the inhibitory effect of tobacco trichome exudates on tobacco blue mold (*Peronospora tabacina*) and on cucumber anthracnose (*Colletotrichum lagenarium*), and showed that cembranoids had antifungal activity. Ferchmin et al. [12] demonstrated that cembranoids from tobacco and corals could affect mouse neuronal-type nicotinic acetylcholine receptors; therefore, cembranoids and their derivatives might have important uses in terms of smoking cessation and the treatment of nicotine addiction. Related research found that 2,7,11-CBT-4,6-diol possessed extensive antimicrobial [13,14,15,16,17], antioxidant, and antitumor activities [15,18,19,20,21]. The present research further enriches our knowledge of the breadth of tobacco cembranoids biological activity, and provides a research foundation for new uses of tobacco.

In the present study, tobacco cembranoids at 80 μg/mL could completely inhibit the growth of *V.* *mali*, and the EC_50_ value is 13.18 μg/mL. Wang et al. [22] showed that the inhibitory rate of 0.8 mg/mL *Polygonum cuspidatum* extract against *V. mali* was 96.5%. Polyhydroxy dinaphthaldehyde isolated from plants could effectively inhibit the growth of *V. mali* at 50.0 mg/mL, and the EC_50_ value is 25.04 mg/mL [23]. These inhibitory rates are far lower than that of tobacco cembranoids extract. The high inhibitory effect of tobacco cembranoids extract against *V. mali* indicates that it has considerable potential to be developed as a biological pesticide to control apple canker (rot). Thus, the effects of tobacco cembranoids extract against *V. mali* should be tested in the field.

According to several reports [8,24,25], the secretion of cembranoids in tobacco trichomes is controlled by two genes and the secretion of trichomes is positively correlated with its density in tobacco varieties, which is consistent with the results of this study. Wagner [10] reported that tobacco TI1068 could secrete 139.5 kg cembranoids per hectare. Severson et al. [9] reported that fresh tobacco leaves from strains such as TI606 could generate 151 μg cembranoids per square centimeter. Therefore, tobacco appears to be a rich resource for generating cembranoids.

According to the results of our study, the content of cembranoids in tobacco flowers is significantly higher than that in leaves. Therefore, tobacco flowers represent a rich resource for cembranoids and can be used as raw material for their extraction. China is the top tobacco-producing country, accounting for 41.5% of the total world tobacco production. Notably, for agricultural tobacco production, the plant is required to be decapitated in the flowering stage, and the tobacco flowers are discarded material in cigarette production. Therefore, this study provides a basis for utilization of tobacco waste generated during agricultural production.

### 2.5. Preliminary Mechanic Exploration of Tobacco Cembranoids Antifungal Activity

#### 2.5.1. Effect of Tobacco Cembranoids on the Content of Ergosterol in the *V. mali* Cell Membrane

**Figure 6:**
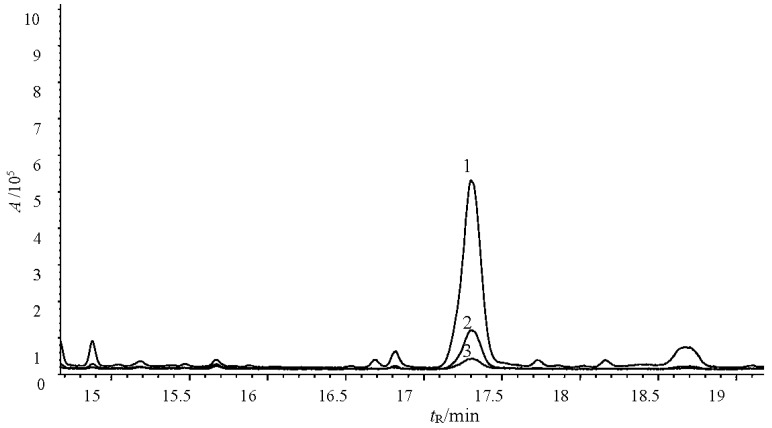
Ergosterol content of untreated and treated *V. mali* hyphal cell membranes. Peak 1: The content of ergosterol in membranes treated with 40 μg/mL (working concentration) tobacco cembranoids extract; Peak 2: Ergosterol content following 20 μg/mL (working concentration) treatment; Peak 3: Ergosterol content following treatment 95% ethanol (control).

Ergosterol is the characteristic component in the filamentous fungal cell membrane [26], and increased ergosterol content in the cell membrane can lead to membrane thickening. Figure 6 shows the fungal cell membrane ergosterol content after treatment with different concentrations of tobacco cembranoids extract or 95% ethanol (control treatment). 

We found that the ergosterol content was higher in the fungi treated with extract than that with control treatment, and that treatment with higher tobacco cembranoids concentration extract could cause higher ergosterol content. These results suggested that the fungal hyphal cell membrane should become thicker after treatment with tobacco cembranoids extract.

#### 2.5.2. Ultrastructural Observation of *V. mali* by Scanning Electron Microscopy

**Figure 7:**
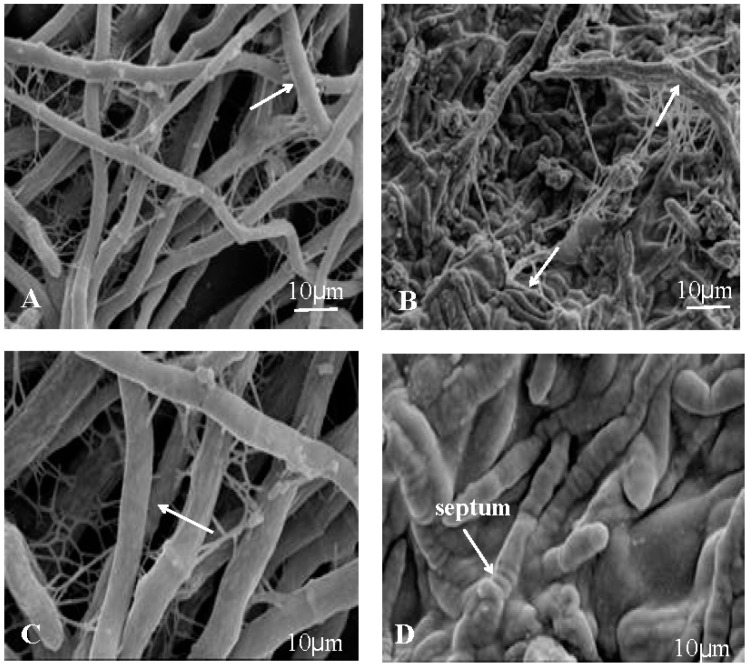
Scanning electron microscopy images of untreated and treated *V. mali* hyphal structure. The untreated hypha appeared round and plump, with structural integrity. (**A**) 800×; (**C**) 3000×. After treatment with 40 μg/mL (working concentration) cembranoids extract, the hypha was deformed, showing adhesion and obvious septa. (**B**) 800×; (**D**) 3000×.

Figure 7A,C show that the nutrient mycelium of control was round and plump, with structural integrity and uniform distribution. However, after treatment with 40 μg/mL (working concentration) tobacco cembranoids extract, the fungi lost their normal morphological structure, with the substrate mycelium shrunk and adhesion, and surface depression and abnormal mycelium with septum were also observed (Figure 7B,D).

#### 2.5.3. Ultrastructural Observation of *V. mali* with Transmission Electron Microscopy

**Figure 8:**
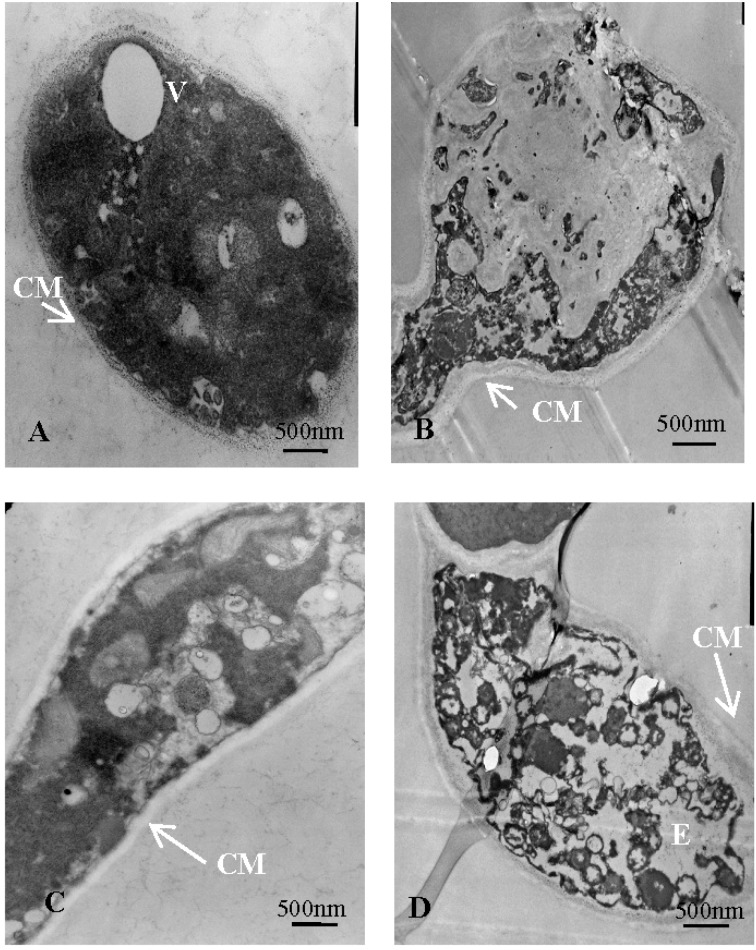
Transmission electron microscopy images of untreated and treated *V. mali* hyphal structures (× 10 k). (**A**) Horizontal and (**C**) vertical sections of untreated hyphal structures show regular, uniform cytoplasm; (**B**) Horizontal and (**D**) vertical sections of structures following 40 μg/mL (working concentration) cembranoids extract treatment. It can be observed that the cell wall became distorted, the plasma membrane was unevenly thickened, and the intracellular composition was disordered.

Figure 8A,C show that the untreated mycelial cross-sectional shape was nearly oval, possessed regular and clear structure, and that the cytoplasm was uniform. However, after treatment with 40 μg/mL (working concentration) tobacco cembranoids extract, the fungi became deformed, the organelles were degraded, the vacuole was ruptured, the intracellular cavity had increased, and the membrane was unevenly thickened (Figure 8B,D). These results were consistent with the ergosterol content result.

Previous studies on the antibacterial mechanism of terpene compounds showed that after treatment with terpenes, the bacterial cell membrane was thickened, suggesting that the mechanism of antibacterial action might be through the concomitant interference with bacterial cell division [27]. This phenomenon is similar to our findings regarding fungal membrane thickening and Yan et al. [16] demonstrated that α-cembratriendiol substantially altered the expression of *V. mali*’*s* genes involved in the redox process, tetrapyrrole binding, coenzyme binding, heme binding, and iron binding; therefore, it can be speculated that cembranoids might also interfere with the fungal cell division process.

Furthermore, we found that the structure of treated fungal cells was damaged severely (Figure 8B,D), so cembranoids might also destroy the selective permeability of the tested fungal membrane. In future studies, we will analyze this possibility using fluorescence labeling.

## 3. Materials and Methods

### 3.1. Materials

The plant materials for this study included seven tobacco varieties, i.e., NC82, CF87, FC8, V2, B22, Jingyehuang, and Gexin NO.3. All of these materials were obtained from the China Tobacco Germplasm Platform (Qingdao, China). The tested fungal strain *V. mali* was purchased from the College of Plant Protection (Northwest A&F University, Yangling, China) and stored in a −80 °C freezer. A pure single cell culture was activated on potato dextrose agar (PDA) medium. After 3 days of culture in the dark at 25 °C, a fungal cake of 5 mm diameter from the edge of the colony was inoculated on PDA medium to obtain a sufficient amount of *V. mali* for subsequent experiments.

### 3.2. Tobacco Field Cultivation

Tobacco materials were planted under field condition using conventional cultivation methods at the Jimo experimental field of Tobacco Research Institute of Chinese Academy of Agricultural Sciences, locating in Shandong Province. Each tobacco cultivar was planted in 10 rows with a distance of 1.2 m, and 25 plants were grown in each row with a 0.5 m space.

### 3.3. Preparation of Tobacco Samples 

At the tobacco flowering stage, flowers and 2–3 leaves in the middle position were collected from 100 plants of each cultivar. The collected tobacco materials were dried in a blast drier (Blue Sky Laboratory Instrument Factory, Hangzhou, China) at 45 °C, and then ground into powder with a grinder mill to pass through a 0.37 mm sieve.

### 3.4. Chemical Compounds Extraction and Isolation

Sample of the pulverized tobacco material (50 g) was weighed and then immersed in 500 mL of extraction solvent, i.e., *n*-hexane, petroleum ether (30–60 °C), 95% ethanol, 80% ethanol, anhydrous ethanol, methanol, ethyl acetate, acetone, or methylene chloride (Sinopharm Chemical Reagent Co., Ltd., Beijing, China). After maintaining at room temperature (30 ± 2 °C) for 24 h, the sample was treated with ultrasonic-assistant extraction for 30 min at 40 °C and filtrated to collect the supernatant. The residue was extracted with same amount of solvent twice more. Then, the extracts were combined to be dried with a freeze-dryer (Alpha 1–2 LD Plus; Christ, Osterode am Harz, Germany), and dissolved in 100 mL of 95% ethanol for further fungicide activity.

### 3.5. Preparation of Tobacco Cembranoids and Content Determination

The inflorescence of flue-cured variety CF87 was subjected to 2000 mL dichloromethane extraction in a glass beaker for 2 seconds, and then subjected to two additional extractions in beakers containing fresh dichloromethane. The extraction was collected and replaced with fresh solvent for further extraction when it changed to cinnamon in color. Then, the extracts were combined to be dehydrated with anhydrous sodium sulfate, and filtered with filter paper. The filtrate was then evaporated under vacuum at 40 °C. Approximately 8 g of crude extracts could be obtained in one extraction. Two g of the extract was added into 10 mL of 70% ethanol containing 0.2 g of activated carbon, and subjected to an ultrasonic bath for 10 min at room temperature. After centrifuging for 10 min at 1210 g, the supernatant underwent vacuum filtration, and the sediment was washed three times with 10 mL 70% ethanol. The resulting supernatant was combined and concentrated to 25–30 mL under vacuum at 50 °C, to which 10 mL of distilled water was added. Then, the mixture was extracted four times with petroleum ether (volume ratio 1:1), and the petroleum ether layer was combined and evaporated to dry under vacuum at 45 °C to yield ~1.25 g of pale-yellow cembranoids extract. The extract was dissolved and diluted with dichloromethane, and its purity was determined using gas chromatography/mass spectrometry (GC/MS) using an Agilent 7890A gas chromatograph coupled with an Agilent 5975C mass selective detector (Agilent Technologies, Santa Clara, CA, USA). The column was an Agilent HP-5MS (30 m × 250 μm × 0.25 μm film thickness), and the injection volume was 1 μL. The carrier gas was helium at a flow rate of 1.2 mL/min in split mode (20:1). The oven and injector temperature was maintained at 280 °C, while the chromatographic column temperature of the GC was programmed with an initial temperature of 80 °C (maintained for 1 min), then increased at the rate of 15 °C/min to 250 °C (maintained for 1 min), and finally increased at the rate of 30 °C/min to 290 °C (maintained for 10 min). The MS detector was operated in scan mode (mass range 40–550 amu); the ionization voltage was 70 eV, the MS source temperature was 230 °C, and the MS Quadruple temperature was 150 °C. Cembranoids were identified by comparing the MS fragmentation patterns with those of reference compounds in the NIST database. Quantification was based on the area under each cembranoid peak as compared to the total area of all cembranoids peaks.

### 3.6. Assessment of Fungicidal Activity

The fungicidal activity of tobacco extracts was determined by the growth-rate method [26]. Under sterile conditions, the extract (1 mL) was added to 100 mL sterilized PDA medium (Land Bridge Technology Co. Ltd., Beijing, China) when it cooled down to about 45 °C, and poured into petri dishes (15 mL per dish) after mixing by shaking. Medium added an equal volume of 95% ethanol was used for control experiment. And, each extract was tested in triplicate. After solidification, the medium was inoculated with 6.0 mm diameter mycelium/agar cubes of *V. mali*. The plates were placed at 25 °C in a constant temperature incubator (SPX-80BSH-II, CIMO, Shanghai, China), and the colony diameter was measured when that grown on the control plate reached about 2/3 of the plate diameter. The inhibition percentage was calculated with following formula [28]: % Inhibition = (Colony diameter of control − Colony diameter of sample) × 100/Colony diameter of control, in which Colony diameter (mm) = Measured value − 6.0.

### 3.7. Determination of the Fungicide Activity of Tobacco Cembranoids Extracts

The tobacco cembranoids extracts were serially diluted in prepare solutions containing 8, 4, 2, 1, 0.5, and 0.3 mg/mL of extracts, respectively. The inhibition rates of different dilution on *V. mali* growth were determined as above described. The logarithm of the concentration of cembranoids extracts were taken as the horizontal axis, and the inhibition rate probability value as the vertical axis to determine the toxicity regression equations, and the concentration of the extract was calculated when its inhibitory rate on the tested fungi was 50% (EC_50_).

### 3.8. Observation of V. mali Ultrastructure

#### 3.8.1. Observation of *V. mali* Ultrastructure by Scanning Electron Microscopy

A 0.5 cm^3^ cube of the fungal mycilium was cut off from the control and treated culture of *V. mali* as described above. Samples were fixed in 2% glutaraldehyde for 2–4 h. After rinsing, the samples were dehydrated with serial ethanol, transferred into isoamyl acetate for 30 min, then dried by CO_2_ critical point drying, stuck and coated by ion sputtering, and finally observed using JSM-840 scanning electron microscopy (JEOL Corporation, Tokyo, Japan) [29].

#### 3.8.2. Observation of *V. mali* Ultrastructure by Transmission Electron Microscopy

Samples collection and fixation was performed as described above. After immobilized in osmic acid, the samples were dehydrated with serial ethanol solutions. Then, the samples were transferred into acetone and penetrated with soak solution. Following standard procedure, the samples were embedded in resin, and sectioned into thin slices. After double-stained with uranyl acetate and lead nitrate, the sections were observed with JEM-1200EX transmission electron microscopy (JEOL Corporation, Tokyo, Japan) [26]. 

### 3.9. Determination of the Main Fungicide Components in the Tobacco Extracts

The concentration of polyphenols in the extracts was determined by high performance liquid chromatography according to the method of Wang et al. [30] with slight modification, and each sample was tested in triplicate. The extract was diluted with 50% methanol to a suitable concentration, and then filtered with a 0.22-μm organic filter membrane into LC ampoules. Samples (5 μL) were injected into a WATERS-ACQUITY HPLC (Waters Corp., Milford, MA, USA) equipped with a UV-VIS spectrophotometer detector. Separations were performed on a BEH C18 column (2.1 mm × 100 mm, 1.7 μm particle size) and the chromatograms were recorded at 340 nm. Polyphenol compounds were analyzed in standard and sample solutions using a gradient elution at 0.3 mL/min with the following gradient program: initial solution of 15% A, then 35% A at 5 min, 80% A at 7 min, 100% A at 9 min, 60% A at 11 min, and 15% A at 14 min. The mobile phase comprised methanol as solvent A and acetic acid: water (1:99) as solvent B. The quantitative analysis of the components was achieved with reference to the authentic standards chlorogenic acid and rutin, which were purchased from Sigma-Aldrich (St. Louis, MO, USA). Component identification was carried out by comparing the UV absorption spectra and retention times of each compound with those of the standards injected under the same condition. The compounds were quantified using calibration curves of standard compounds as the mean of three replicates.

The concentration of solanesol was determined by high performance liquid chromatography (Waters) according to the method of Liu et al. [31], all assays were run in triplicates. The extract (0.2 mL) was saponified in a centrifuge tube by using 0.1 M ethanol solution of sodium hydroxide (1 mL) as the saponifier, and the saponification reaction was carried out with ultrasonic-assistance at a temperature of 40 °C, ultrasonic frequency of 45 KHz, and the time was 30 min after the saponification was finished, following which the centrifuge tube and saponified extract was allowed to cool to room temperature. To this, *n*-hexane (5 mL) and deionized water (8 mL) were added, followed by centrifugation for 10 min after being thoroughly shaken. The upper *n*-hexane (0.5 mL) was pipetted out and diluted with the mobile phase to 5 mL, and then the solution was filtered with a 0.22-μm organic filter membrane into LC ampoules and analyzed using WATERS-ACQUITY HPLC equipped with a UV-VIS spectrophotometer detector. Separation of the target compound was performed on an ACQUITY UPLC BEH C18 column (1.7 mm × 5 mm, 1.7 μm particle size) using methanol as the mobile phase by isocratic elution. Flow rate was set at 0.3 mL/min, column temperature was 35 °C, and injection volume was 2 μL. The quantitative wavelength of the UV detector was set at 208 nm. Quantitative analysis of the target compound was achieved with reference to an authentic standard of solanesol (>95%, TCI Corporation, Tokyo, Japan).

The concentration of terpenoids were determined by Agilent 7890A gas chromatograph coupled with an Agilent 5975C mass selective detector (Agilent Technologies) according to the method of Severson et al. [32] with slight modification, all assays were run in triplicates. The extract (0.5 mL) was injected into a tube and dried with a nitrogen-blowing instrument. To this, exactly 5 mL dichloromethane and deionized water was added. The sample was then centrifuged for 10 min after being thoroughly shaken. The lower dichloromethane was pipetted out and filtered with a 0.22-μm organic filter membrane. The sample (1 μL) was injected into the DB-5MS capillary column (30 m × 250 μm × 0.25 μm) with split mode injection and a split ratio at 2:1. The injection temperature was maintained at 280 °C, while the chromatographic column temperature of the GC was programmed with an initial temperature of 120 °C (maintained for 4 min), then increased at the rate of 30 °C/min to 230 °C (maintained for 1 min), further increased at the rate of 15 °C/min to 250 °C (maintained for 1 min), and finally increased at the rate of 30 °C/min to 280 °C (maintained for 14 min). Helium was used as the carrier gas system with a flow rate of 1.2 mL/min. The GC/MS interface temperature was maintained at 230 °C, and the energy of the ion source was 70 eV. The quadrupole temperature was maintained at 150 °C. The terpenoids were identified based on the comparison of the MS fragmentation patterns with those of reference compounds in the NIST database and the retention time of the standards, and quantified using the internal standard method.

### 3.10. Determination of the Ergosterol Content in the Cell Membrane of V. mali

According to the method of Gao et al. [33], the *V. mali* was cultured on PDA in a thermostatic incubator (25.5 °C). After 3 days, four fungal pieces with consistent and vigorous growth were placed in 50 mL PD. After 4 days of cultivation with shaking (140 r/min, 25.5 °C), 0.5 mL extract of different concentration was added (or 95% ethanol as a control) to concentrations of 40 or 20 μg/mL. After culturing for another 3 days, the hyphae were collected and washed several times with 10 mL of 25 mM phosphate buffer (pH 7.4) until the supernatant was colorless; they were then centrifuged (TDZ5-WS, Xiangyi, Changsha, China) for 10 min at 1210 *g* to collect the treated mycelium. Then, 2.5 g of sample was weighed out, ground in liquid nitrogen, mixed with 3 mL of methanol/dichloromethane (*v*:*v* = 2:1) solvent, and incubated for 1 h at 25 °C. Subsequently, 5 mL water, 10 mL dichloromethane, and 5 mL 0.5 M phosphate buffer (pH 7.4; containing 2.0 M KCl) were added in turn. After stratification, the methylene chloride phase was collected and dried in water bath (60 °C). The dry residue was saponified in 5 mL methanol/ethanol (*v*:*v* = 4:1) solvent containing 1.4 M KOH for 1 h at 60 °C before adding 5 mL water and 10 mL petroleum ether (boiling range 60–90 °C). The petroleum ether layer was collected after stratification and evaporated to dryness. The dry matter was then dissolved in hexane and brought to a constant volume of 3 mL. Each treatment was repeated in triplicate.

An authentic standard of ergosterol was obtained from China National Medicines Corporation Ltd., (Beijing, China). The ergosterol content was subjected to GC/MS analysis in an Agilent 7890A gas chromatograph coupled with an Agilent 5975C mass selective detector (Agilent Technologies), in triplicate. The sample (2 μL) was injected into the DB-5MS capillary column (30 m × 250 μm × 0.25 μm) with split mode injection and a split ratio at 1:1. The injection temperature was maintained at 250 °C, while the chromatographic column temperature of the GC was programmed with an initial temperature of 200 °C (maintained for 1 min), then increased at the rate of 8 °C/min until 290 °C (maintained for 10 min). Helium was used as the carrier gas system with a flow rate of 1 mL/min. The ergosterol was identified based on the comparison of the MS fragmentation patterns with those of reference compounds in the NIST database and the retention time of the standard, and the difference of the content of ergosterol in the *V. mali* cell membrane between different cembranoids extracts concentration treatments was obtained by comparing the ergosterol peak areas.

### 3.11. Statistical Analysis

Data are reported as the means ± SD of three measurements. Data Processing System (DPS) 7.05 was used for the data statistics and the regression analysis of the experimental data. Statistical significance was determined by one-way ANOVA followed by Duncan’s multiple range method. *p*-value less than 0.05 was considered to be significant.

## 4. Conclusions

Our study examined the in vitro inhibitory effect of tobacco extract on *V. mali* and determined the effective antifungal chemical constituents therein. We demonstrated that tobacco extracts obtained using non-polar or less-polar organic solvents had a higher inhibitory effect on *V. mali*. Furthermore, tobacco cembranoids were found to be the key effective antifungal components in the extracts, and their antifungal mechanism might occur primarily through destroying the structure of the hyphal internal membrane to inhibit the growth of the mycelium. Due to the differences in cembranoids content in the different tobacco varieties and between the flowers and leaves of same variety, the antifungal activities differed significantly between different tobacco extracts. This research provides a basis for the application of cembranoids as a plant-sourced fungicide, and for the selection of a highly efficient cembranoids resource. In addition, the agricultural production of tobacco requires decapitation in the flowering stage, which makes tobacco flowers as waste materials whose surface is rich of cembranoids. These observations in this study suggest that tobacco flower has good potential to be a readily available and inexpensive natural source of efficient biological pesticides for controlling apple canker.

## Figures and Tables

**Table 1:** Virulence of tobacco cembranoids extract against the hyphal growth of *V.* *mali*.

Treatment	Regression Equation	R^2^	*p*-Value	MIC (μg/mL)	MFC (μg/mL)	EC_50_ (μg/mL)
Tobacco cembranoids extract	y = 1.5923x + 3.2114	0.97	0.003	2.02	80	13.18

## Abbreviations

The following abbreviations are used in this manuscript:

MIC	minimal inhibition concentration
MFC	minimal fungicidal concentration
EC_50_	half-maximal effective concentration
GC/MS	gas chromatography/mass spectrometry
HPMS	high pressure mass spectrometry
HPLC	high performance liquid chromatography
CM	cytoplasmic membrane
V	vacuole
E	cavum
